# Podocyte apoptosis is prevented by blocking the Toll-like receptor pathway

**DOI:** 10.1038/cddis.2015.125

**Published:** 2015-05-07

**Authors:** P Saurus, S Kuusela, E Lehtonen, M E Hyvönen, M Ristola, C L Fogarty, J Tienari, M I Lassenius, C Forsblom, M Lehto, M A Saleem, P-H Groop, H Holthöfer, S Lehtonen

**Affiliations:** 1 Department of Pathology, University of Helsinki, Helsinki, Finland; 2 Laboratory Animal Centre, University of Helsinki, Helsinki, Finland; 3 Folkhälsan Research Center, Folkhälsan Institute of Genetics, Helsinki, Finland; 4 Division of Nephrology, Helsinki University Central Hospital, Helsinki, Finland; 5 Diabetes and Obesity Research Program, Research Program's Unit, University of Helsinki, Helsinki, Finland; 6 Department of Pathology, HUSLAB and Helsinki University Hospital, Helsinki and Hyvinkää, Finland; 7 Bristol Royal Hospital for Children, University of Bristol, Bristol, UK; 8 Baker IDI Heart and Diabetes Institute, Melbourne, Australia; 9 Department of Bacteriology and Immunology, University of Helsinki, Helsinki, Finland

## Abstract

High serum lipopolysaccharide (LPS) activity in normoalbuminuric patients with type 1 diabetes (T1D) predicts the progression of diabetic nephropathy (DN), but the mechanisms behind this remain unclear. We observed that treatment of cultured human podocytes with sera from normoalbuminuric T1D patients with high LPS activity downregulated 3-phosphoinositide-dependent kinase-1 (PDK1), an activator of the Akt cell survival pathway, and induced apoptosis. Knockdown of PDK1 in cultured human podocytes inhibited antiapoptotic Akt pathway, stimulated proapoptotic p38 MAPK pathway, and increased apoptosis demonstrating an antiapoptotic role for PDK1 in podocytes. Interestingly, PDK1 was downregulated in the glomeruli of diabetic rats and patients with type 2 diabetes before the onset of proteinuria, further suggesting that reduced expression of PDK1 associates with podocyte injury and development of DN. Treatment of podocytes *in vitro* and mice *in vivo* with LPS reduced PDK1 expression and induced apoptosis, which were prevented by inhibiting the Toll-like receptor (TLR) signaling pathway with the immunomodulatory agent GIT27. Our data show that LPS downregulates the cell survival factor PDK1 and induces podocyte apoptosis, and that blocking the TLR pathway with GIT27 may provide a non-nephrotoxic means to prevent the progression of DN.

Lipopolysaccharides (LPS) are fat-soluble outer membrane components of the Gram-negative bacteria. It has been shown that normoalbuminuric patients with type 1 diabetes (T1D) who progress to microalbuminuria have higher baseline serum LPS activity than normoalbuminuric non-progressors.^1^ This indicates that high LPS activity in sera of patients with T1D is associated with the development of microalbuminuria.^1^ The origin of circulating endotoxins in patients with diabetes is not yet fully known. In addition to severe bacterial infections,^2^ underlying systemic diseases (e.g., periodontitis) and life-style related factors (e.g., high-fat diet) may increase plasma levels of endotoxins in humans.^3, 4, 5^ Interestingly, LPS in the sera of septic patients has previously been shown to induce apoptosis of kidney cells,^6^ but the mechanism is not known.

Podocytes are terminally differentiated and highly specialized cells that are required for normal glomerular function. Podocyte loss due to apoptosis or detachment is a key component of progressive glomerulosclerosis. Podocyte loss has been reported in patients with T1D^7^ and type 2 diabetes (T2D) with or without diabetic nephropathy (DN),^8, 9, 10^ and Verzola *et al.*^8^ reported glomerular apoptosis in the kidneys of patients with DN. In Pima Indians with T2D, decreasing number of podocytes per glomerulus has been shown to be the strongest indicator of the progression of the renal disease.^9^ Podocytes are also detected in the urine in patients with diabetes, and podocyte number in urine correlates with the severity of the disease.^10^ These data indicate that analysis of the regulation of apoptosis in podocytes is essential for better understanding of the pathophysiological mechanisms of glomerular diseases.

The central role of the phosphoinositide 3-kinase (PI3K)-dependent Akt signaling pathway in the regulation of cell survival raises the molecules that modulate its activity to key roles in regulating apoptosis in podocytes. 3-Phosphoinositide-dependent protein kinase-1 (PDK1) is a 63-kDa serine/threonine kinase that functions downstream of PI3K but upstream of Akt and serves as a major regulatory point in Akt signaling.^11^ Mice lacking PDK1 die *in utero*, and mice that lack PDK1 specifically in the pancreatic *β* cells develop progressive hyperglycemia as a result of the loss of islet mass.^12, 13^

We hypothesized that PDK1, the key regulator of the PI3K/Akt-mediated cell survival pathway, could have a role in regulating podocyte apoptosis, and that high LPS activity could downregulate PDK1, consequently inducing apoptosis and podocyte injury.

## Results

### PDK1 is expressed in glomerular podocytes

**Figure 1:**
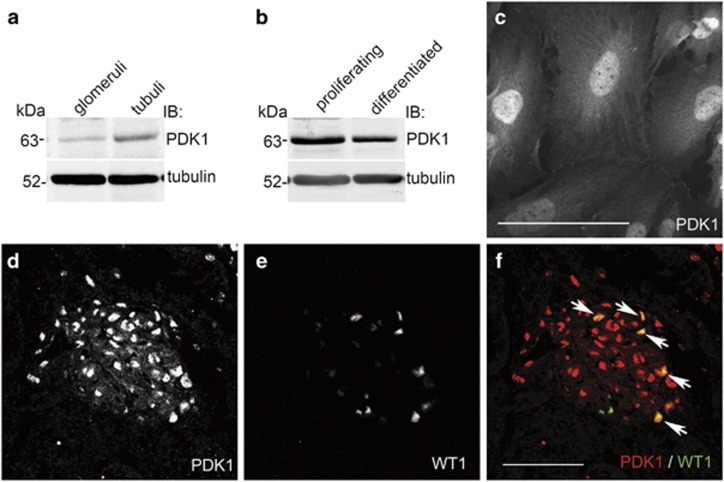
PDK1 is expressed in glomerular podocytes. (**a**) Immunoblot of rat glomerular and tubular fractions shows PDK1 expression in glomeruli and tubuli. Tubulin is included as a loading control. (**b**) Immunoblot of cultured human podocytes shows that PDK1 is expressed in both proliferating and differentiated podocytes. Tubulin is included as a loading control. (**c**) Immunofluorescence microscopy indicates that PDK1 localizes in both nuclei and cytoplasm in differentiated podocytes. (**d**–**f**) Rat kidney sections stained for PDK1 (**d**) and WT1 (**e**) show that PDK1 localizes in both nuclei (arrows) and cytoplasm in podocytes as well as in other glomerular cells as visualized in the merged image (**f**). (**a** and **b**) 70 *μ*g of glomerular, tubular or cultured podocyte lysates were immunoblotted with anti-PDK1 or anti-tubulin IgGs. (**c**) Differentiated human podocytes were fixed with PFA, labeled with anti-PDK1 IgG, and examined by fluorescence microscopy. (**d–f**) Rat kidney cryosections were fixed with acetone, labeled with anti-PDK1 and anti-WT1 IgGs, and examined by confocal microscopy. Scale bars: (**c**–**f**) 50 *μ*m

PDK1 mRNA is expressed ubiquitously in human tissues,^14, 15, 16^ but its localization and function in glomerular podocytes is unknown. Immunoblotting of isolated rat glomerular and tubular fractions revealed that PDK1 is expressed in both glomeruli and tubuli (Figure 1a). PDK1 is also expressed in proliferating and differentiated cultured human podocytes (Figure 1b), and localizes in both nuclei and cytoplasm (Figure 1c). Double labeling of normal rat kidney sections with PDK1 and Wilms tumor 1 (WT1) antibodies confirmed that PDK1 is expressed in the nuclei of podocytes (Figures 1d–f). PDK1 is also detected in other glomerular cells (Figures 1d–f).

### Sera with high LPS activity from T1D patients reduce the expression of PDK1 and induce apoptosis in cultured human podocytes

**Figure 2:**
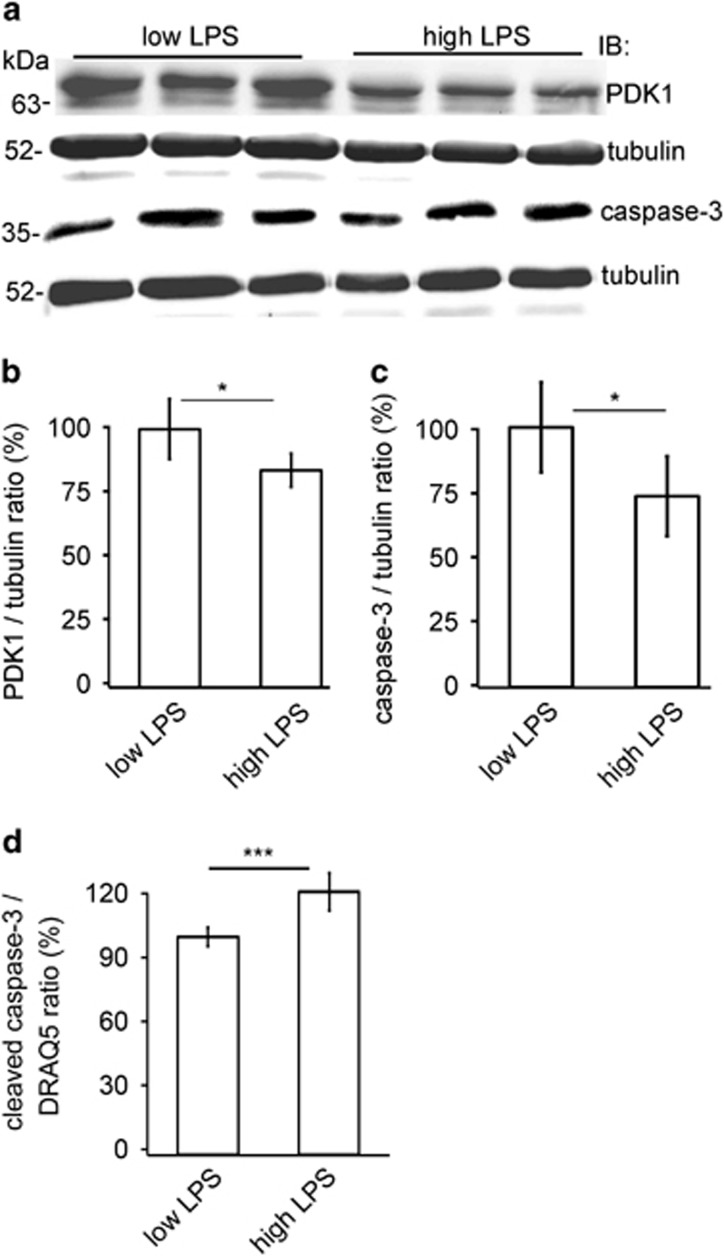
Sera with high LPS activity from normoalbuminuric human patients with T1D reduce the expression of PDK1 and induce apoptosis in cultured human podocytes. (**a**) Representative immunoblot of PDK1 and total caspase-3 in lysates of differentiated human podocytes after 72 h treatment with sera with low or high LPS activity. Tubulin is included as a loading control. (**b** and **c**) Quantification of PDK1 and total caspase-3 from podocytes treated with sera with high or low LPS activity (*n*=6) shows that the expression of PDK1 (**b**) and total caspase-3 (**c**) was lower after treatment with high-LPS sera compared to treatment with low-LPS sera. Caspase-3 is activated by cleavage, and therefore reduced level of total caspase-3 indicates an increase in apoptosis. (**d**) In-Cell Western of cleaved caspase-3 in podocytes treated with sera with high or low LPS activity shows that the expression of cleaved caspase-3 was higher after treatment with high-LPS sera compared to treatment with low-LPS sera. The signal of cleaved caspase-3 was normalized with the nuclear marker DRAQ5. Treatments were performed with sera from individual patients. The experiment was performed three times. The bars (**b**–**d**) show the mean expression in arbitrary units (error bars S.D.). *P<0.05, ***P<0.001, Student's *t*-test.

We found previously that high baseline LPS activity in sera of Finnish patients with T1D, even though normoalbuminuric, is associated with the progression of DN.^1^ This, together with the potential antiapoptotic role of PDK1, led us to hypothesize that serum LPS could reduce the expression of PDK1 and induce podocyte injury and thereby contribute to the development of DN. To test this hypothesis, we subjected cultured podocytes to sera from normoalbuminuric T1D patients with either high or low LPS activity (Supplementary Table S1). PDK1 was downregulated after 72 h exposure to sera with high LPS activity when compared with cells treated with sera with low LPS activity (Figures 2a and b). Sera with high LPS activity also increased apoptosis as indicated by decreased level of total caspase-3 (Figures 2a and c) and increased level of cleaved caspase-3 (Figure 2d). We also tested whether high glucose reduces the expression of PDK1, as it has been previously shown that high glucose induces podocyte apoptosis.^17, 18^ Treatment of human podocytes with high glucose decreased the expression level of PDK1 compared with cells cultured in normal glucose or in high mannitol (osmolality control) (Supplementary Figure S1). Notably, blood glucose values were similar in patients with low and high serum LPS activity (Supplementary Table S1), indicating that high glucose did not contribute to reduced expression or increased apoptosis in this experimental set-up.

### Knockdown of PDK1 increases apoptosis in cultured human podocytes

**Figure 3:**
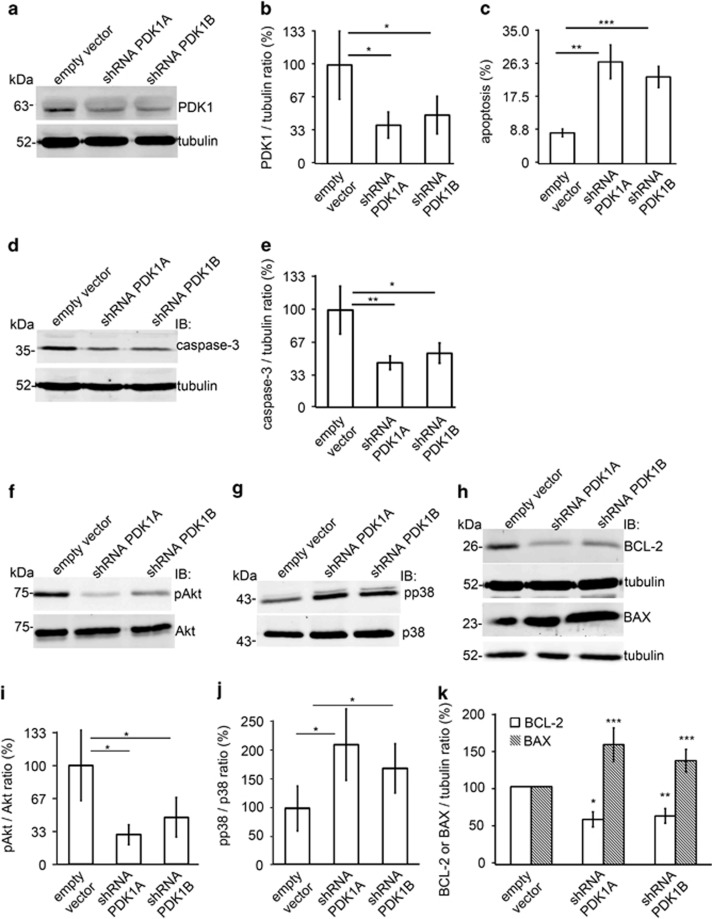
Knockdown of PDK1 by shRNAs increases apoptosis and downregulates antiapoptotic and upregulates pro-apoptotic pathways in cultured human podocytes. (**a**) Western blot analysis of PDK1 knockdown efficiency. Human podocytes were infected using two different shRNA constructs targeting PDK1 (shRNA PDK1A and shRNA PDK1B). Empty vector shRNA served as a control. Tubulin is included as a loading control. (**b**) PDK1 protein level is significantly decreased by both shRNAs compared with empty vector shRNA. (**c**) Flow cytometry of podocytes stained with Annexin V shows that PDK1 knockdown increases podocyte apoptosis. (**d**) Representative immunoblot of total caspase-3 in PDK1 knockdown cells. Tubulin is included as a loading control. (**e**) Quantification of total caspase-3 after PDK1 knockdown shows decreased total caspase-3 expression indicating an increase in apoptosis. (**f**) Representative immunoblotting for phosphorylated S437-Akt (pAkt) in podocytes after PDK1 knockdown with two different shRNA constructs (shRNA PDK1A and shRNA PDK1B). Total Akt is included as a loading control. (**g**) Representative immunoblot of phosphorylated Thr180/Tyr182-p38 (pp38) in PDK1 knockdown podocytes. Unphosphorylated p38 is included as a loading control. (**h**) Representative immunoblot of BCL-2 and BAX in PDK1 knockdown podocytes. Tubulin is included as a loading control. (**i**) Quantification of phosphorylated Akt and total Akt in three replicate analyses as in (**f**) shows that knockdown of PDK1 decreases Akt activity in cultured human podocytes. (**j**) Quantification of p38 and phosphorylated p38 (pp38) MAPK in three replicate analyses as in (**g**) shows that knockdown of PDK1 increases p38 activity in cultured human podocytes. (**k**) Quantification of BCL-2 and BAX in three replicate analyses as in (**h**) shows that the expression level of BCL-2 is downregulated and BAX upregulated after PDK1 knockdown. All experiments were performed three times with three replicates in each experiment. The bars (**b**, **c**, **e**, **i**, **j**, and **k**) show the mean expression in arbitrary units (error bars S.D.). *P<0.05, **P<0.01, ***P<0.001, Student's *t*-test

To confirm that PDK1 protects podocytes against apoptosis, we knocked down PDK1 in cultured podocytes using two different lentiviral small-hairpin RNAs (shRNAs). Both shRNAs lowered PDK1 level compared with podocytes infected with viruses carrying the empty vector (EV) (Figures 3a and b). Fluorescence-activated cell sorting (FACS) of Annexin V-labeled cells indicated that the level of apoptosis was increased from 6% in podocytes infected with the EV to 22–27% in podocytes infected with PDK1 shRNAs (Figure 3c). In untreated control cells, the level of apoptosis was similar to that in cells infected with the EV (4–8% in both), and the number of necrotic cells was not increased by PDK1 knockdown (1–2%). The level of total caspase-3 was lower in podocytes infected with PDK1 shRNAs compared with podocytes infected with the EV (Figures 3d and e) confirming higher rate of apoptosis in PDK1-deficient podocytes.

### Knockdown of PDK1 inhibits antiapoptotic and stimulates proapoptotic pathways

To determine whether apoptosis-related kinases, Akt and p38 MAPK, are involved in the antiapoptotic PDK1 pathway in podocytes, we examined the activation of these pathways after knockdown of PDK1. Knockdown of PDK1 reduced the phosphorylation level of Akt on S437 (Figures 3f and i), and increased the activation of the proapoptotic p38 MAPK pathway by inducing phosphorylation of p38 (Figures 3g and j). There was no difference in Akt and p38 MAPK phosphorylation between control and empty vector-transfected podocytes (data not shown). Depletion of PDK1 also decreased the level of antiapoptotic BCL-2 and increased the level of proapoptotic BAX indicating the involvement of the intrinsic apoptotic pathway (Figures 3h and k).

### PDK1 is downregulated in the glomeruli of diabetic rats and patients with T2D

**Figure 4:**
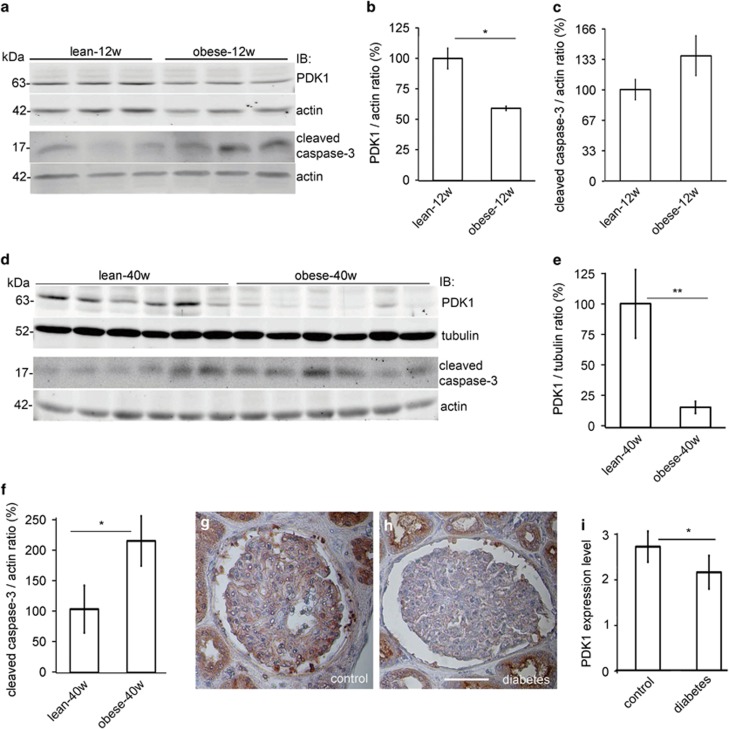
PDK1 expression is downregulated in the glomeruli of obese Zucker rats and in the glomeruli of human patients with T2D. (**a**) The expression level of PDK1 is decreased and cleaved caspase-3 slightly increased in the glomeruli of 12-week-old obese Zucker rats compared with lean controls. Actin is included as a loading control. (**b** and **c**) Quantification of PDK1 and cleaved caspase-3 in the glomeruli of three individual lean and three individual obese 12-week-old Zucker rats. Downregulation of PDK1 is significant whereas the increase in cleaved caspase-3 does not reach significance. (**d**) Immunoblot of PDK1 and cleaved caspase-3 in the glomeruli of 40-week-old obese Zucker rats. Tubulin and actin are included as a loading control. (**e** and **f**) Quantification of PDK1 and cleaved caspase-3 in the glomeruli of six individual lean and six individual obese 40-week-old Zucker rats shows that the expression of PDK1 is lower and cleaved caspase-3 higher in the glomeruli of obese Zucker rats. (**a** and **d**) Glomeruli were isolated, lysed and immunoblotted with anti-PDK1 and anti-cleaved caspase-3 IgGs. (**g** and **h**) PDK1 staining in the glomerulus of control (**g**) and in patient with diabetes (**h**). Human kidney sections were processed for immunoperoxidase staining and labeled with anti-PDK1 IgG. Scale bar: 25 *μ*m. (**i**) Quantification of the staining intensity of PDK1 indicates that the expression of PDK1 is lower in the glomeruli of patients with diabetes than in controls. The staining intensity of PDK1 was graded visually (scale 1–5) in 6 glomeruli of 18 patients with diabetes and 17 controls by two independent researchers blinded from the diabetes status. The bars (**b**, **c**, **e**, **f**, and **i**) show the mean expression in arbitrary units (error bars S.D.). (**b** and **c**) *n*=3. (**e** and **f**) *n*=6. *P<0.05, **P<0.01, Student's *t*-test

As podocyte loss by apoptosis or detachment is an early feature of DN,^9, 17, 19^ and knockdown of PDK1 increased apoptosis in podocytes, we analyzed whether PDK1 expression is reduced in the glomeruli of insulin-resistant obese Zucker rats.^20^ Zucker rats show increased podocyte apoptosis at 21 weeks of age when compared with lean controls,^21^ and develop proteinuria by 40 weeks.^22^ The expression level of PDK1 was lower in the glomeruli of obese rats at 12 (Figures 4a and b) and 40 weeks (Figures 4d and e) compared with lean controls. Apoptosis was not significantly increased in 12-week-old obese rat glomeruli (Figures 4a and c). At 40 weeks, the level of cleaved caspase-3 was increased in the glomeruli of the obese rats, indicating increased glomerular apoptosis (Figures 4d and f).

We also analyzed the expression of PDK1 in the glomeruli of patients with T2D and controls. The patients with diabetes did not have clinical nephropathy, and histopathological analysis revealed no signs of DN. The expression of PDK1 was lower in patients with diabetes compared with controls (Figures 4g–i).

### Inhibition of the TLR pathway prevents LPS-induced downregulation of PDK1 and induction of apoptosis in cultured human podocytes

**Figure 5:**
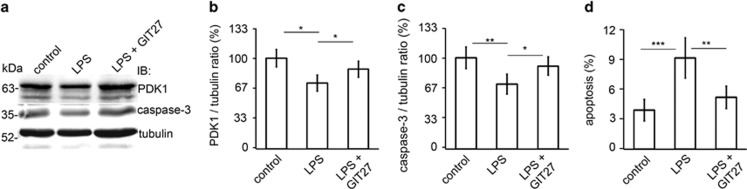
Inhibition of the TLR pathway prevents LPS-induced downregulation of PDK1 and induction of apoptosis in cultured human podocytes. (**a**) Representative immunoblot of PDK1 and total caspase-3 in LPS-treated podocytes with or without GIT27 co-treatment. Tubulin is included as a loading control. Quantification of PDK1 (**b**) and total caspase-3 (**c**) shows that co-treatment of podocytes with LPS and GIT27 prevents downregulation of PDK1 and induction of apoptosis. (**d**) Flow cytometry of podocytes stained for Annexin V confirms that co-treatment of podocytes with GIT27 and LPS prevents induction of apoptosis. The experiment was performed three times with three replicates in each experiment. The bars (**b**–**d**) show the mean expression in arbitrary units (error bars S.D.). *P<0.05, **P<0.01, ***P<0.001, Student's *t*-test

Podocytes express Toll-like receptors (TLRs), including TLR4, which acts as a receptor for LPS.^23, 24, 25^ We hypothesized that inhibition of the TLR pathway could serve as a treatment to reduce LPS-induced downregulation of PDK1 and apoptosis of podocytes. To this end, cultured podocytes were treated with 4,5-Dihydro-3-phenyl-5-isoxazoleacetic acid (GIT27) before addition of LPS to the media. GIT27 is an active immunomodulatory agent that inhibits TLR4 and TLR2/6 signaling pathways. Co-treatment of podocytes with GIT27 and LPS prevented downregulation of PDK1 and induction of podocyte apoptosis induced by LPS as determined by immunoblotting for caspase-3 and FACS analysis of Annexin V-labeled cells (Figures 5a–d).

### Inhibition of the TLR pathway prevents LPS-induced downregulation of glomerular PDK1 and induction of tubular apoptosis in BALB-C mice

**Figure 6:**
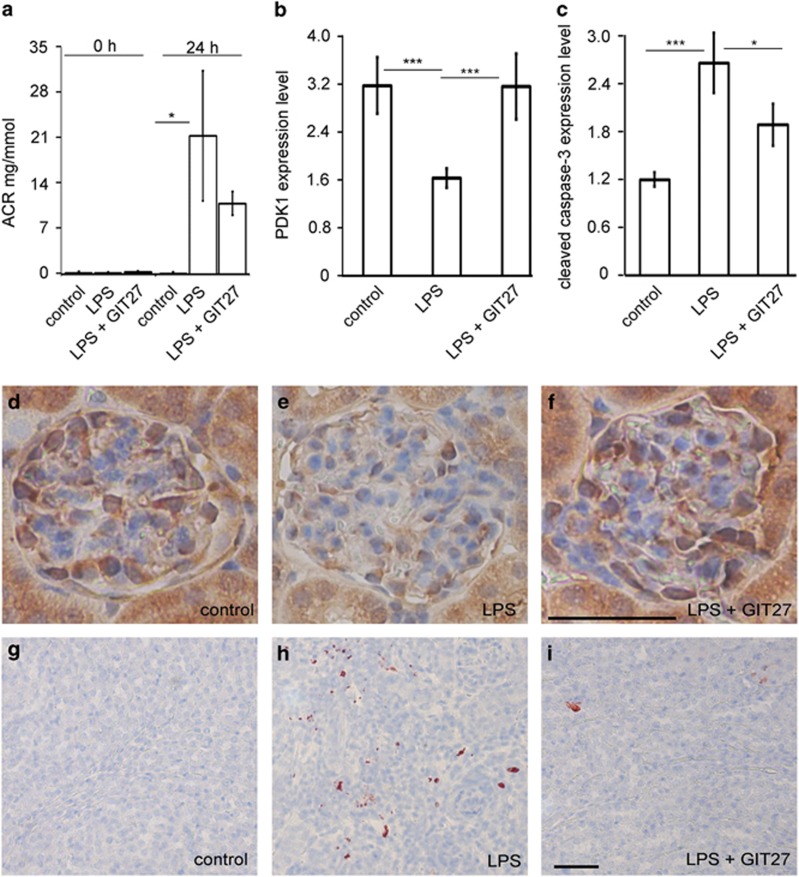
Inhibition of the TLR pathway prevents LPS-induced downregulation of PDK1 and induction of apoptosis in BALB-C mouse kidneys. (**a**) Effects of LPS and GIT27 on urinary albumin excretion in experimental animals. Spot urine samples were collected before (0 h) and 24 h after LPS challenge. Albumin-to-creatinine ratio (ACR) indicates that LPS induces statistically significant albuminuria that is partially prevented by co-treatment with GIT27, but this did not reach statistical significance. (**b**) Quantification of the staining intensity of PDK1 indicates that the expression of PDK1 is lower in the glomeruli of LPS-treated mice than in controls. Co-treatment of mice with LPS and GIT27 prevents downregulation of PDK1. (**c**) Quantification of the staining intensity of cleaved caspase-3 indicates increased apoptosis in kidneys of LPS-treated mice. Co-treatment with GIT27 prevents LPS-induced apoptosis. (**d**–**f**) Examples of PDK1 staining in control (**d**), LPS-treated (**e**), and LPS- and GIT27-treated (**f**) mouse glomeruli. Scale bar (**d**–**f**) 20 *μ*m. (**g**–**i**) Examples of cleaved caspase-3 staining in control (**g**), LPS-treated (**h**), and LPS- and GIT27-treated (**i**) mouse kidneys. Scale bar (**g**–**i**) 20 *μ*m. (**d**–**i**) Mouse kidney paraffin sections were processed for immunoperoxidase staining and labeled with anti-PDK1 or anti-cleaved caspase-3 IgGs. The staining intensities of PDK1 and cleaved caspase-3 were rated visually (scale 1–5) in six glomeruli (**b**) or random fields of kidney cortex (**c**) of each mouse by two independent researchers blinded from the treatment. Analysis was made of six controls, six LPS-treated, and six LPS- and GIT27-treated mice. *P<0.05, ***P<0.001, Students's *t*-test, error bars S.D.

To study whether LPS reduces the expression of PDK1 *in vivo* and whether reduction in PDK1 expression can be blocked by the inhibition of the TLR pathway similarly as *in vitro*, we used LPS to induce podocyte injury and proteinuria in BALB-C mice. Mice were treated with either GIT27 or its vehicle 24 h before and 2 h after LPS challenge. Albuminuria, PDK1 expression and kidney morphology were analyzed 24 h after the LPS challenge. LPS increased urinary albumin excretion (Figure 6a), decreased glomerular expression of PDK1 (Figures 6b, d and e), and increased tubular apoptosis (Figures 6c, g and h). GIT27 treatment prevented the decrease in the expression of PDK1 (Figures 6b, d and f) and the increase in apoptosis (Figures 6c, g and i). The effect on urinary albumin excretion rate was not statistically significant (Figure 6a).

### Inhibition of the TLR pathway prevents LPS-induced podocyte foot process widening and reduction in slit diaphragm protein podocin in BALB-C mice

**Figure 7:**
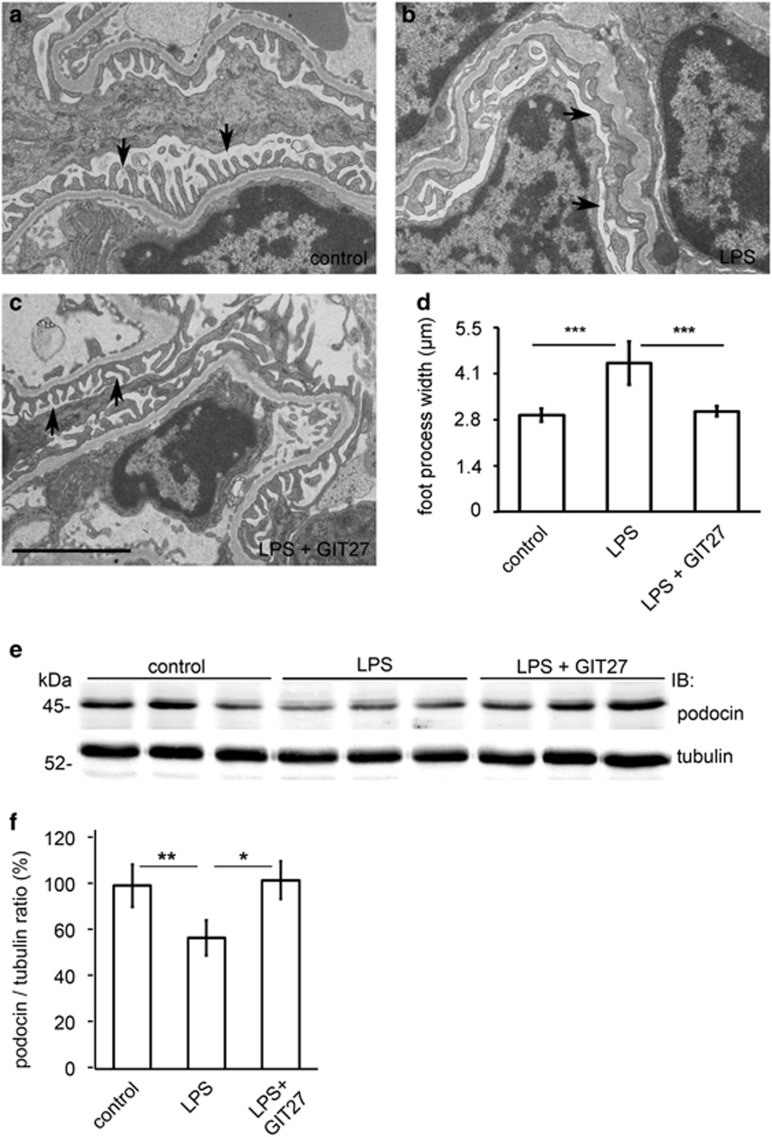
Inhibition of the TLR pathway prevents LPS-induced podocyte foot process effacement and downregulation of slit diaphragm protein podocin. (**a**) Electron microscopy of control mouse kidney reveals that podocyte foot processes line regularly the glomerular basement membrane around capillary loops. (**b**) Twenty-four hours LPS treatment in mice induces podocyte foot process widening. (**c**) Co-treatment of mice with GIT27 and LPS prevents LPS-induced podocyte foot process widening. Arrows, podocyte foot processes. (**d**) Quantification of podocyte foot process width confirms that LPS causes foot process widening, which is prevented by GIT27 pretreatment. Foot process width was calculated from 3 to 4 animals per group, 3 glomeruli per animal, and 3 capillary loops per glomeruli. (**e**) Representative immunoblot of podocin in control, LPS-treated and LPS- and GIT27-treated mouse kidneys. Tubulin is included as a loading control. (**f**) Quantification of podocin from control mouse kidneys and kidneys treated with LPS with or without GIT27 treatment. Analysis was made of six controls, six LPS- treated, and six LPS- and GIT27-treated mice. *P<0.05, **P<0.01, ***P<0.001, Student's *t*-test, error bars S.D. Scale bar: 2 *μ*m

Mice were treated with LPS and GIT27 as described above. Electron microscopy (EM) revealed that podocyte foot processes were wider in the LPS-treated mice when compared with control mice and mice treated with LPS and GIT27 (Figures 7a–d). Level of slit diaphragm protein podocin, which is essential for podocyte survival,^26^ was also reduced in LPS-treated mice and this was prevented by TLR blockade (Figures 7e and f). The data indicate that inhibition of the TLR pathway in BALB-C mice can prevent LPS-induced podocyte foot process widening and reduction in podocin expression level.

### Inhibition of the TLR pathway prevents downregulation of PDK1 and induction of podocyte apoptosis induced by sera from T1D patients with high LPS activity

**Figure 8:**
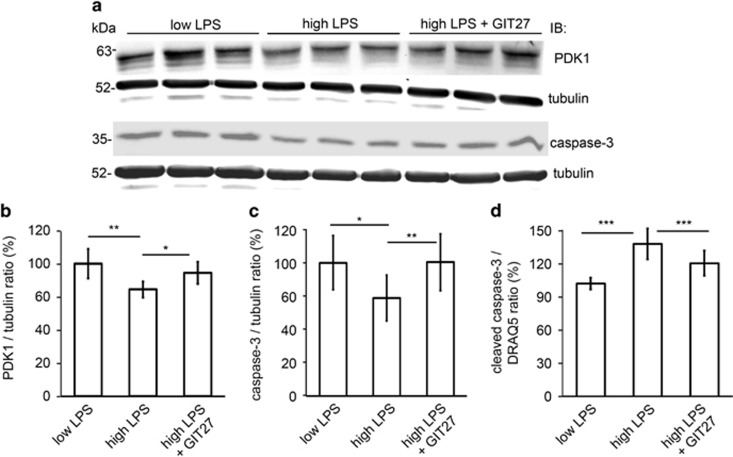
Inhibition of the TLR pathway prevents downregulation of PDK1 and induction of apoptosis induced in cultured human podocytes by 72 h treatment with human sera with high LPS activity. (**a**) Representative immunoblot for PDK1 and total caspase-3 in cultured podocytes treated with sera with low or high LPS activity, and with or without GIT27 co-treatment. Tubulin is included as a loading control. (**b** and **c**) Quantification of PDK1 and total caspase-3 from podocytes treated with sera with high or low LPS activity (*n*=6 each), or treated with sera with high LPS activity in the presence of GIT27 (*n*=6). The expression of PDK1 (**b**) and total caspase-3 (**c**) was lower after treatment with sera with high LPS activity than with low LPS activity. GIT27-treatment prevented the downregulation of PDK1 (**b**) and the induction of apoptosis (**c**) induced by high LPS activity. (**d**) In-Cell Western of cleaved caspase-3 in podocytes treated with sera with high or low LPS activity with or without GIT27 treatment shows that the expression of cleaved caspase-3 was higher after treatment with high-LPS sera compared to treatment with low-LPS sera and that GIT27-treatment prevented the induction of apoptosis. The signal of cleaved caspase-3 was normalized with DRAQ5. Treatments were performed with sera from individual patients. The experiment was performed three times with three replicates in each experiment. The bars (**b**–**d**) show the mean expression in arbitrary units (error bars S.D.). *P<0.05, **P<0.01, ***P<0.001, Student's *t*-test

To confirm that downregulation of PDK1 is caused by LPS, cultured podocytes were treated with GIT27 for 2 h before supplementing the media with sera from normoalbuminuric T1D patients with high LPS activity. The expression level of PDK1 was downregulated when podocytes were exposed to the sera with high LPS activity compared with exposure to the sera with low LPS activity (Figures 8a and b). In podocytes pretreated with GIT27, PDK1 expression remained higher (Figures 8a and b), indicating that high LPS activity downregulates PDK1 expression in podocytes, and this can be prevented by blocking the receptor of LPS. Treatment with GIT27 before the addition of sera with high LPS activity was also able to reduce podocyte apoptosis induced by high LPS activity (Figures 8a, c and d). The data suggest that inhibition of the TLR signaling pathway by GIT27 has therapeutic potential for treating DN.

## Discussion

Long-term treatment of mice with LPS leads to metabolic endotoxemia causing increased general inflammation and diabetes.^27^ LPS also induces albuminuria in mice.^28, 29^ Our previous study shows that high serum LPS activity in patients with T1D associates with the progression of DN.^1^ This may be due to increased inflammation as chronic inflammation also contributes to the development of DN.^30^ In support of these observations, we have also found that the incidence of bacterial infections correlates with the severity of diabetic nephropathy in Finnish patients with T1D.^2^ Here, we show that treatment of cultured podocytes with sera from T1D patients with high LPS activity reduces the expression of PDK1, the key mediator of the PI3K/Akt cell survival pathway, and increases apoptosis. We further show that inhibition of the TLR signaling pathways, initiated by binding of LPS to its receptor TLR4, prevents the LPS-induced reduction in the expression of PDK1 both *in vitro* and *in vivo*. This suggests that inhibiting the cellular activities of LPS by blocking the TLR signaling pathways provides a means to reduce podocyte loss and thereby prevent the progression of DN.

LPS induces apoptosis in various cell types, especially in endothelial cells,^31, 32^ in the kidney, and also specifically in podocytes.^33, 34, 35^ The mechanism by which LPS induces apoptosis is incompletely understood, but caspase inhibition has been shown to protect against LPS-induced apoptosis.^34, 36^ The observation that LPS downregulates PDK1 provides a novel mechanistic insight by which LPS induces apoptosis in podocytes. The antiapoptotic role of PDK1 was confirmed by knocking PDK1 down in podocytes, which led to an increase in apoptosis. Reduced level of PDK1 stimulated the proapoptotic p38 MAPK pathway and increased the expression of BAX, and reduced the activity of the PI3K/Akt cell survival pathway and the expression of BCL-2. BCL-2 and BAX have also previously been shown to be involved in podocyte apoptosis.^37, 38^ The expression of BAX and active caspase-3 was found to be elevated in diabetic rat glomeruli and in cultured mouse podocytes treated with high glucose.^38^ Here, we show that PDK1 is concentrated in the nuclei of podocytes, and nuclear PDK1 has been shown to suppress cellular apoptosis.^39^ Our data showing a role for PDK1 as an antiapoptotic protein is further supported by the finding that inhibition of the Akt pathway with a specific PDK1 inhibitor induces apoptosis.^40^ In line with this, induction of PDK1 activity decreases apoptosis by activating Akt.^11^ Supporting a protective role for PDK1 in the kidney *in vivo*, we observed that PDK1 was downregulated in the glomeruli of obese Zucker rats, a model of T2D that shows similarities to early human DN,^22^ already at an early stage before the rats developed significant proteinuria or glomerular apoptosis. PDK1 was also downregulated in the glomeruli of patients with T2D who did not yet have clinical nephropathy and after treatment with high glucose. These data suggest that downregulation of PDK1 contributes to podocyte injury and may thus be involved in the development of DN.

Administration of LPS to mice *in vivo* reduced the level of PDK1 in the glomeruli and induced apoptosis in the kidney tubules. We were unable to detect apoptosis in LPS-treated mouse glomeruli by immunohistochemical staining for active caspase-3. This may be due to technical limitations, as detaching cells are hard to visualize. However, a previous study supports our data, by showing that LPS treatment leads to approximately 15% reduction in the number of podocytes and upregulation of genes involved in apoptosis.^35^ In Gram-negative sepsis, LPS induces apoptosis of tubular cells leading to acute renal failure,^41^ and treatment of cultured podocytes or kidney tubular cells with sera from septic patients also increases apoptosis.^6^ In line with the above, we also observed increased apoptosis of tubular cells in LPS-treated mouse kidneys. As PDK1 is expressed in kidney tubules, it is possible that PDK1 is also involved in the regulation of tubular apoptosis and acute renal failure. This, however, requires further studies.

Podocytes express TLR4 that acts as a receptor for LPS.^23, 24, 25^ It has been previously shown that TLR4 is necessary for LPS responsiveness, since both C3H/HeJ and C57BL/10ScCr mice, in which TLR4 gene has been mutated, are low responders to LPS.^25, 42^ Hoshino *et al.*^43^ demonstrated that mice lacking TLR4 are hyporesponsive to LPS.^43^ Of note, Jialal *et al.*^44^ have recently shown that global TLR4 deficiency in streptozotocin-induced diabetic mice (C57BL/6J) inhibits development of renal inflammation, fibrosis, and podocytopathy.^44^ It can therefore be postulated that inhibiting the activity of the TLR signaling pathway could block the effects of LPS on podocytes. Indeed, we found that in both podocytes *in vitro* and mice *in vivo*, treatment with the TLR pathway inhibitor GIT27 prevented the effects of LPS by maintaining the expression levels of PDK1 and podocin and preventing apoptosis and foot process widening. In mice, GIT27 seemed to reduce LPS-induced albuminuria although this did not reach statistical significance due to high individual variation in the LPS treatment group. The protective effect of GIT27 on renal function in T2D is supported by a previous study in diabetic db/db mice, where long-term GIT27 administration decreased albuminuria and glomerulosclerosis.^45^ The clinical significance of our data is corroborated by the observation that in cultured podocytes, downregulation of PDK1 and increase in apoptosis, induced by T1D patient sera with high LPS activity, was prevented by GIT27. Since GIT27 inhibitis TLR4 and TLR2,^46^ both shown to be expressed in podocytes^23^ and linked with diabetes,^47, 48, 49^ the protective effect of GIT27 treatment might be due to inhibition of pathways initiated via both TLR4 and TLR2. Further support for a protective role of GIT27 in T1D comes from studies with animal models indicating that GIT27 reduces the cytokine-mediated immunoinflammatory events that destruct the pancreatic islets.^50^

The data above point out that the blockade of the TLR pathway has potential in the treatment of DN. The TLR signaling pathway blocker GIT27 may have clinical potential and is very attractive, as, unlike the well-known antibiotic with high LPS-binding capacity, polymyxin b sulfate,^51^ GIT27 seems not to be nephrotoxic.^45, 52^ Furthermore, both *i.p* and *p.o.* administration of GIT27 are equally efficient in preventing streptozotocin-induced diabetes.^50^ However, the wide expression of TLRs in various tissues warrants further studies to define the tissue-specific effects of GIT27 or other TLR pathway effectors when developing new TLR-targeting therapies.

Collectively, this study shows that PDK1 is one of the downstream effectors of the endotoxin LPS. Preventing downregulation of PDK1 by inhibiting the TLR path-way may protect podocytes against injury and loss by apoptosis.

## Materials and Methods

### Animal studies

Glomeruli were isolated by sieving^53^ from male Sprague-Dawley or obese (fa/fa) and lean (fa/+) Zucker rats (Crl:ZUC-Leprfa, Charles River Laboratories, Sulzfeld, Germany). Blood glucose and albuminuria of the Zucker rats have been described previously.^54^

Seven-week-old female BALB-C mice (BALB/cAnNCrl) (Scanbur, Karlsunde, Denmark) were treated with LPS (Sigma-Aldrich, St. Louis, MO, USA) and GIT27 (Tocris Bioscience, Bristol, UK), LPS and PBS or PBS only (*n*=6 in each group). Spot urine samples were obtained at baseline. Mice were injected intraperitoneally with GIT27 (20 mg/kg) or equal volume of PBS. After 24 h, LPS (12 mg/kg) was administered intraperitoneally. Control mice received PBS. Another dose of GIT27 or PBS was injected 2 h after LPS administration. Twenty-four hours after LPS injection, spot urine samples were collected, mice were killed and kidneys processed for analysis. Urinary albumin and creatinine analyses were carried out at the Biochemical Analysis Core for Experimental Research (http://www.biomed.helsinki.fi/research/services/bacer/) at the Institute of Biomedicine, University of Helsinki with ADVIA 1650 Chemistry System (Siemens AG Healthcare, Erlangen, Germany) according to the manufacturer's instructions. Mice and rats were maintained according to the principles of laboratory animal care, and the experiments were approved by the National Animal Experiment Board.

### Cell culture

Conditionally immortalized human podocytes were cultured under standard conditions.^55^ Shortly, cells were maintained at +33 °C in RPMI media supplemented with 10% fetal bovine serum (FBS), 1% glutamine, and insulin, transferrin and sodium selenite (ITS). To induce differentiation, podocytes were transferred to +37 °C for 14 days. Media, FBS, and ITS were obtained from Sigma-Aldrich and ultraglutamine from Lonza (Basel, Switzerland).

### Immunofluorescence microscopy

Differentiated podocytes were fixed with 2% paraformaldehyde (Electron Microscopy Sciences, Hatfield, PA, USA) in PBS and permeabilized with 0.1% Triton X-100 in PBS. Rat kidney cryosections were fixed with acetone. Samples were blocked with CAS-block (Invitrogen, Carlsbad, CA, USA), incubated with rabbit anti-PDK1 (Cell Signaling Technology, Danvers, MA, USA) and mouse anti-WT1 IgGs (Upstate, New York, NY, USA) for 1 h at room temperature (cells) or overnight at +4 °C (tissue sections) in ChemMate (Dako Cytomation, Glostrup, Denmark), followed by AlexaFluor 555 donkey anti-rabbit (Invitrogen) or DyLight 488 donkey anti-mouse IgGs (Jackson Immuno Research Laboratories Inc., West Grove, PA, USA), and Hoechst (Fluka, Sigma-Aldrich) for 1 h in ChemMate (Dako Cytomation). Samples were mounted in Mowiol and examined with Zeiss Axiophot 2 microscope (Carl Zeiss Microscopy GmbH, Jena, Germany) or Leica SP2 confocal microscope (Leica Microsystems CMS GmbH, Mannheim, Germany).

### Immunoblotting

Immunoblotting was performed as in Hyvonen *et al.*^54^ Membranes were incubated overnight at +4 °C with rabbit anti-PDK1, rabbit anti-p38 MAPK, rabbit anti-phospho-p38 MAPK (Thr180/Tyr182), rabbit anti-phopho-Akt (Ser473), mouse anti-caspase-3 or rabbit anti-cleaved caspase-3 (Cell Signaling Technology), mouse anti-Pan Akt (R&D Systems, Minneapolis, MN, USA), rabbit anti-Bax or rabbit anti-Bcl2 (Abcam, Cambridge, UK), mouse anti-tubulin or mouse anti-actin IgGs (Sigma-Aldrich), followed by Alexa Fluor 680 (Invitrogen) or IRDye 800 (LI-COR, Lincoln, NE, USA) donkey anti-rabbit, anti-goat or anti-mouse IgGs. Detection and quantification was performed with an Odyssey Infrared Imager (LI-COR).

### Treatment of podocytes with sera from patients with diabetes

Male patients with T1D were recruited and examined by the Finnish Diabetic Nephropathy Study (FinnDiane; www.finndiane.fi). Serum LPS activities were determined in 39 T1D patients with normal urinary albumin excretion (AER <30 mg/24 h) as described earlier.^1^ Serum samples with the highest (*n*=6) and lowest (*n*=6) LPS activities were selected for the treatment of podocytes (Supplementary Table S1). Differentiated human podocytes were treated with 10% human sera for 72 h. GIT27 (Tocris Bioscience) (10 *μ*g/ml) was added to the media 2 h before addition of human sera with high LPS activity. The expression levels of PDK1 and caspase-3 were analyzed by immunoblotting as described above. In-Cell Western for cleaved caspase-3 is described below. The use of human material was approved by the local Ethics Committee.

### Knockdown of PDK1 by lentiviral infection

Lentiviral human pLKO1-shPDK1 vectors shPDK1A (GAAGGTATATTAGGACATTTG) and shPDK1B (TATAGACTCAGAAGGTATATT) (Biomedicum Genomics, University of Helsinki, Finland) were used to knock down PDK1 in differentiated human podocytes and an empty pLKO1 vector was used as a control. For virus production, CMVDelta8.9 and phCMVg packaging plasmids, together with shPDK1A, shPDK1B or pLKO1, were transfected into HEK293FT cells (Invitrogen) with Lipofectamine2000 (Invitrogen). Virus-containing media were collected 72 h later, filtrated through 0.45 *μ*m filter, and ultracentrifuged 85 000 × *g* at +4 °C for 90 min. Viruses were resuspended in PBS, added to podocytes on day 10 or 11 of differentiation and incubated at +37 °C for 10 min, followed by centrifugation 1360 × *g* at +4 °C for 30 min. Virus-containing medium was replaced with regular medium after 24 h.

### Immunohistochemistry

Kidney samples of renal cancer patients with or without T2D were obtained from surgical nephrectomies performed at Helsinki and Uusimaa Hospital district, and were from the non-malignant part of the kidney. The diabetes status of the patients was determined from the medical records. The mean age of controls was 74.3 (±5.9) years and patients with diabetes 73.9 (±4.5) years (*P*=0.89, Student's *t*-test). The group of patients with diabetes included 11 men and 7 women, and the control group included 11 men and 6 women. The use of human material was approved by the local Ethics Committee.

Human and mouse kidney samples were fixed with formaldehyde and embedded in paraffin. Deparaffinized sections were stained with anti-PDK1 or cleaved caspase-3 IgGs followed by detection with VectaStain Elite ABC kit (Vector Laboratories, Burlingame, CA, USA) and AEC (Dako Cytomation). Slides were counterstained with hematoxylin and photographed using Nikon Eclipse 800 microscope (Nikon Instruments Europe BV, Amsterdam, Netherlands) using the same microscope settings throughout the analysis.

### Induction and detection of apoptosis

Differentiated podocytes were exposed to LPS (100 ng/ml *Escherichia coli* 0111:B4 LPS (Sigma-Aldrich)) for 48 h. The activity of 100 ng/ml LPS in cell culture media was measured as described,^1^ and was shown to be 1.7 EU/ml. When indicated, GIT27 (Tocris Bioscience) (10 *μ*g/ml) was added to the cells 2 h before LPS exposure. Apoptosis was detected by FACS using Annexin V-FITC Kit and double staining with 7-AAD (BD, Franklin Lakes, NJ, USA) using FACSAria (BD). Cells positive for Annexin V-FITC and negative for 7-AAD were deemed apoptotic. A total of 1 × 10^4^ cells were detected by FACS in each sample. Apoptosis was also detected by immunoblotting for total or cleaved caspase-3 as described above.

For In-Cell Western, podocytes were cultured in black 96-well plates (Perkin-Elmer, Waltham, MA, USA), fixed with 4% paraformaldehyde (Electron Microscopy Sciences, Hatfield, PA, USA) in PBS and permeabilized with 0.1% Triton X-100 in PBS. Cells were incubated with rabbit anti-cleaved caspase-3 (Abcam) at room temperature for 1 h, followed by IRDye 800 (LI-COR) donkey anti-rabbit IgG and 1 *μ*M DRAQ5 (Thermo Fisher Scientific, Waltham, MA, USA) incubation at room temperature for 1 h. Detection and quantification was performed with an Odyssey Infrared Imager (LI-COR). The signal for cleaved caspase-3 was normalized with DRAQ5.

### Electron microscopy

Mouse kidney samples were processed for EM as in Wasik *et al.*^56^ Samples were examined with JEM-1400 Transmission Electron Microscope (Jeol, Tokyo, Japan) equipped with Olympus-SIS Morada digital camera (Olympus Soft Imaging Solutions GmbH, Münster, Germany). Foot process width was determined as described.^57^ Briefly, the number of foot processes per capillary loop was counted, divided by the length of the glomerular basement membrane (GBM), and multiplied by *π*/4. Calculations were performed on 3–4 animals per group, 3 glomeruli per animal and the foot process width was expressed as averages of measurements of 3 capillary loops per glomerulus.

### Statistical methods

All variables were presented as mean±S.D. Statistical differences between groups were determined with the Student's *t*-test (Microsoft, v.2010, Redmond, WA, USA). For all statistics, *P*-values of <0.05 were considered as statistically significant.

## Abbreviations

DN: diabetic nephropathy
EM: electron microscopy
EV: empty vector
FACS: fluorescence-activated cell sorting
FBS: fetal bovine serum
GBM: glomerular basement membrane
GIT27: 4,5-Dihydro-3-phenyl-5-isoxazoleacetic acid
ITS: insulin transferrin and sodium selenite
LPS: lipopolysaccharides
PDK1: 3-phosphoinositide dependent protein kinase-1
PI3K: phosphoinositide 3-kinase
T1D: type 1 diabetes
T2D: type 2 diabetes
TLR: toll-like receptor
WT1: Wilms tumor 1
