# Supplementary material for: Podocyte apoptosis is prevented by blocking the Toll-like receptor pathway
Source: Cell Death Dis. 2015 May 7;6(5):e1752–. doi: 10.1038/cddis.2015.125 (PMC4669704; doi:10.1038/cddis.2015.125)
Supplement: Supplementary Table S1 [file cddis2015125x1.pdf]

Supplementary Table S1. Clinical characteristics of normoalbuminuric T1D patients with low or high serum LPS activity. Normoalbuminuria was defined as urinary albumin excretion rate <30 mg/24 h. All patients were males. HbA1c, hemoglobin A1c; LPS, lipopolysaccharide; Duration, duration of diabetes.

LPS	Low (n=6)	High (n=6)
Age (years)	34.8 ± 3.3	33.2 ± 6.7
Duration (years)	13.8 ± 2.7	14.2 ± 3.9
HbA1c (%)	7.9 ± 0.8	7.7 ± 0.2
Triglycerides (mmol/L)	1.0 ± 0.9	1.2 ± 0.6
LPS (EU/ml)	0.20 ± 0.01	0.41 ± 0.04*
Glucose (mmol/L)	8.63 ± 2.19	5.83 ± 1.08

* $p < 0.001$, Student's t-test.
