# Supplementary material for: Podocyte apoptosis is prevented by blocking the Toll-like receptor pathway
Source: Cell Death Dis. 2015 May 7;6(5):e1752–. doi: 10.1038/cddis.2015.125 (PMC4669704; doi:10.1038/cddis.2015.125)
Supplement: Supplementary Figure S1 [file cddis2015125x2.pdf]

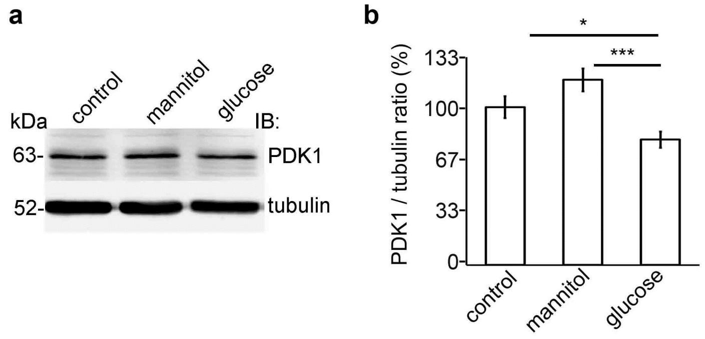

Supplementary Figure S1 PDK1 expression is downregulated in human podocytes treated with high glucose. (**a**) Representative immunoblot of PDK1 expression in control human podocytes (11 mM glucose) and podocytes treated with 30 mM mannitol or 30 mM glucose for 2 weeks. Tubulin is included as a loading control. (**b**) Quantification of PDK1 shows that the expression of PDK1 is lower after high glucose treatment. The experiment was performed three times with three replicates. The bars (**b**) show the mean expression in arbitrary units (error bars STDEV). *$p < 0.05$, ***$p < 0.001$, Student's *t*-test.
